# Case Report: Recurrent Malignant Struma Ovarii With Hyperthyroidism and Metastases, A Rare Case Report and Review of the Literature

**DOI:** 10.3389/pore.2022.1610221

**Published:** 2022-05-10

**Authors:** Ruyi Zhang, Xin Tian, Ying Luo, Haiwei Dong, Weijun Tian, Yujie Zhang, Dong Li, Haoran Sun, Zhaowei Meng

**Affiliations:** ^1^ Department of Nuclear Medicine, Tianjin Medical University General Hospital, Tianjin, China; ^2^ Department of Obstetrics and Gynecology, Tianjin Medical University General Hospital, Tianjin, China; ^3^ Department of General Surgery, Tianjin Medical University General Hospital, Tianjin, China; ^4^ Department of Pathology, Tianjin Medical University General Hospital, Tianjin, China; ^5^ Department of Medical Imaging, Tianjin Medical University General Hospital, Tianjin, China

**Keywords:** case report, surgery, struma ovarii, hyperthyroidism, CA125, thyroglobulin

## Abstract

**Background:** SO (Struma ovarii) is a rare form of ovarian teratoma which originates from ovarian dermoid cysts. Due to the rarity of this disease, relevant studies might not be sufficiently documented, especially cases with hyperthyroidism and multiple metastases.

**Case Presentation:** A 40-year-old female patient was admitted to our hospital due to management of early pregnancy along with a recurrent abdominal and pelvic mass. Contrast-enhanced CT images showed an irregular mass (10.7 × 8.6 × 12.8 cm) located in the right side from the hypogastrium to the pelvic cavity and another mass (3.8 × 3.7 cm) in the liver. Laboratory examination showed that CA125 (Carbohydrate Antigen-125) was 118.10 U/mL, Tg (thyroglobulin) was >300 ng/ml, FT4 (free thyroxine) was 22.11 pmol/L, and TSH (thyroid-stimulating hormone) was <0.004 mIU/L. She subsequently underwent liver mass dissection, omentectomy, tumor dissection, peritoneal nodule resection, as well as rectal anterior wall nodule resection. The patient was diagnosed with malignant SO (papillary type) along with multiple metastases. Also, we conducted a literature review based on 290 SO cases from 257 articles.

**Conclusion:** This study showed that malignant SO might be prone to relapse and metastasize (a metastatic rate of 52.94%) and therefore aggressive management might need to be recommended for malignant SO. Also, laparotomy might need to be recommended for large tumors that cannot be resected by laparoscopic surgery since these tumors might be prone to rupture and thus produce peritoneal implants. Furthermore, Graves’ disease might need to be considered in the differential diagnosis.

## Introduction

SO (Struma ovarii), a rare form of ovarian teratoma which originates (more than 50%) from ovarian dermoid cysts consisting of thyroid tissue, accounts for about 1% of all ovarian neoplasms and about 2% of mature ovarian teratomas (1, 2). According to several reports, the age at diagnosis ranged largely from 12 to 77 years of age with a median age of 43 (3, 4). Due to the rarity of this disease, relevant studies might not be sufficiently documented. Thus, the prognosis and modalities of treatment are still somewhat controversial. This case report presented a rare diagnosis of malignant SO with hyperthyroidism and multiple metastases.

The differential diagnosis between malignant SO and benign SO varied largely over the years. Some researchers hypothesize that malignancy can only be diagnosed when the tumor shows definite invasion or metastases, while others diagnose malignancy on nuclear or histologic alterations (5). In most instances, the diagnosis of malignant SO is only made postoperatively (6). As a result, some cases might have been misdiagnosed as benign SO. At the same time, due to the rarity of this disease, an approved consensus on the diagnosis of benign and malignant SO has not been arrived at yet. In this study, we also retrieved a total of 290 cases from 257 articles aiming to find and discuss more disparities that might be helpful in the differential diagnosis between these two forms of disease.

## Case Presentation

A 30-year-old female with early pregnancy and a left ovarian mass was admitted to a local primary hospital, where she later received a laparoscopic mass dissection and induced abortion in January 2008. Postoperative histology suggested the presence of an inclusion cyst. In August 2008, the patient was readmitted to that hospital due to a right ovarian mass with the greatest dimension up to 10 cm. Then, the patient received another laparoscopic resection of the mass which ruptured in the pelvic cavity during the dissection. Postoperative histology confirmed that the mass was composed of SO (unknown histology, unknown if malignant). The patient did not have any follow-up until 5 years later when a new pelvic cystic mass (4.0 cm in greatest dimension) was discovered. However, she declined treatment. Then in April 2018, at the age of 40, the patient was admitted to the obstetrics and gynecology department of our hospital for management of another early pregnancy along with a recurrent abdominal and pelvic mass which had enlarged from 4.0 to 9.0 cm.

During the physical examination, there were no remarkable findings, apart from a palpable mass in the right lower abdomen with no tenderness or rebound tenderness.

**FIGURE 1:**
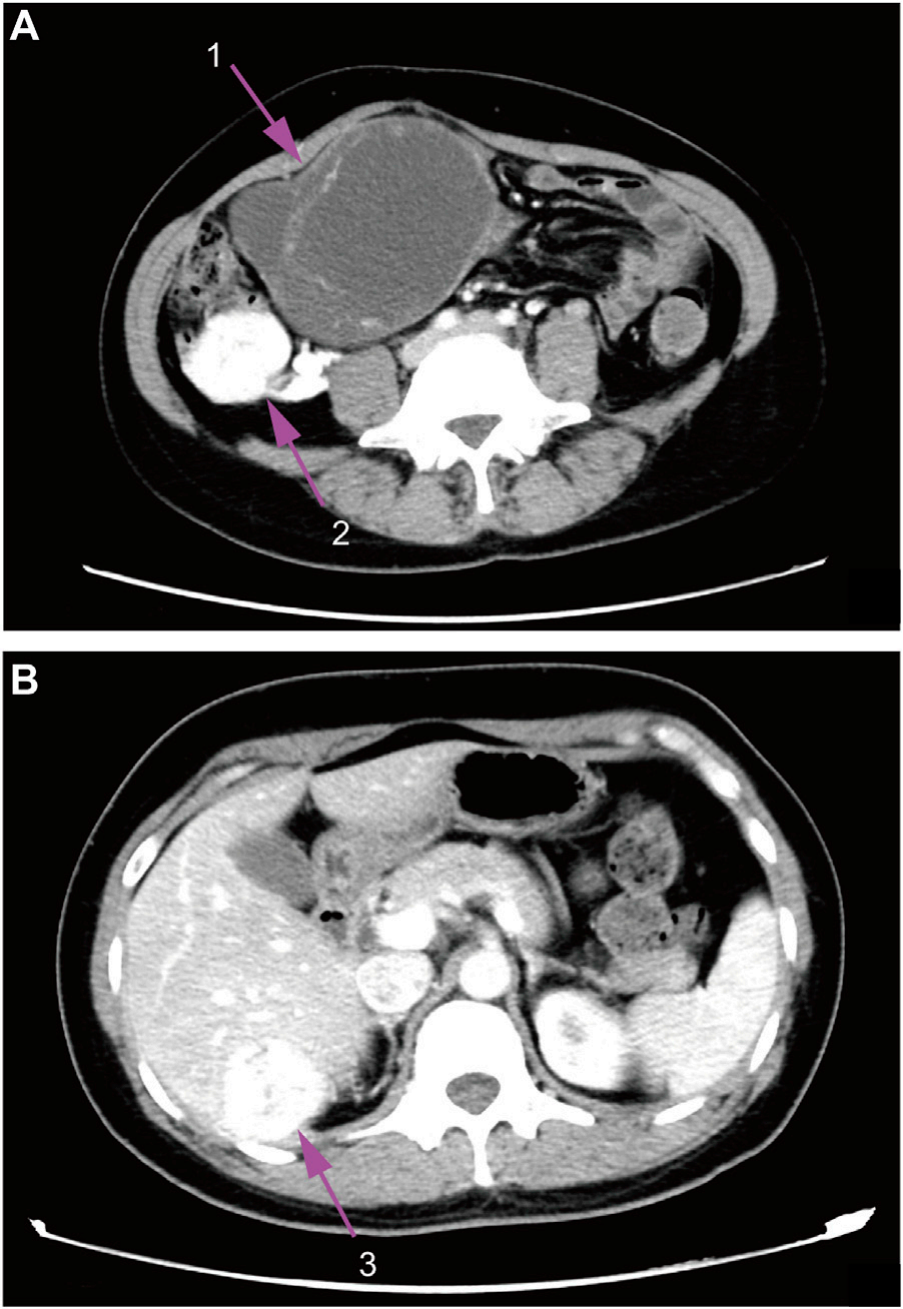
Contrast-enhanced CT images. **(A)**, abdomen image. **(B)**, pelvic cavity image; 1, ovarian mass; 2, surrounding lesions of the ovarian mass; 3, enhancing liver mass.

Contrast-enhanced CT (computed tomography) images of the whole abdomen and pelvic cavity showed an irregular mass (10.7 × 8.6 × 12.8 cm) located in the right side from the hypogastrium to the pelvic cavity containing both solid and cystic components with regional irregular calcifications. The mass was surrounded by multiple enhancing lesions and low-density nodules of different sizes with the largest one up to 4.3 cm in diameter (Figure 1A). In addition, a similar enhancing mass (3.8 × 3.7 cm) was also found in the Couinaud’s S6 segment of the liver (Figure 1B). Gynecological ultrasonography confirmed the abdomino-pelvic mass which contained solid and cystic components. Doppler vascular flow was noted only in the solid component. No abnormality was detected on ultrasonography of the thyroid gland and cervical lymph nodes. Laboratory examination showed that CA125 (Carbohydrate Antigen-125) was 118.10 U/mL (reference range: 0.00–35.00 U/mL), Tg (thyroglobulin) was >300 ng/ml (reference range: 0.00–55.00 ng/ml), FT4 (free thyroxine) was 22.11 pmol/L (reference range: 9.01–19.05 pmol/L), and TSH (thyroid-stimulating hormone) was <0.004 mIU/L (reference range: 0.350–4.940 mIU/L). Carcinoembryonic antigen, alpha-fetoprotein, anti-thyroglobulin antibody as well as calcitonin were all within the normal range. The patient later opted for a surgical abortion to further remove the abdominal and pelvic mass. In May 2018, the patient was transferred to the general surgical department of our hospital to receive surgery.

Laparoscopy showed multiple light red nodules which were each about 1.0 cm in diameter in the greater omentum and the right side of the peritoneum, one large exophytic mass (5.0 cm in diameter) with a grey surface in the S6 segment of the liver (without invasion of the liver parenchyma), as well as a large polycystic mass in the right adnexal region with a high-surface-tension and a complete capsule. No ascites had formed in the patient’s abdomen. A diagnostic resection of the peritoneal nodules was performed using an ultrasonic scalpel and tissues were immediately sent for rapid pathological examination. It showed that components of thyroid tissue could be found in the nodules and thus they were considered to be metastatic lesions that might arise from the SO. Subsequently, a liver mass dissection and omentectomy were performed in sequence. Laparoscopic instruments were then withdrawn. Laparotomy was immediately performed. During the laparotomy, total tumor dissection, unilateral salpingo-oophorectomy, peritoneal nodule resection, as well as rectal anterior wall nodule resection were performed. Due to the patient’s strong will of preserving her fertility, the uterus and the left ovary were preserved.

**FIGURE 2:**
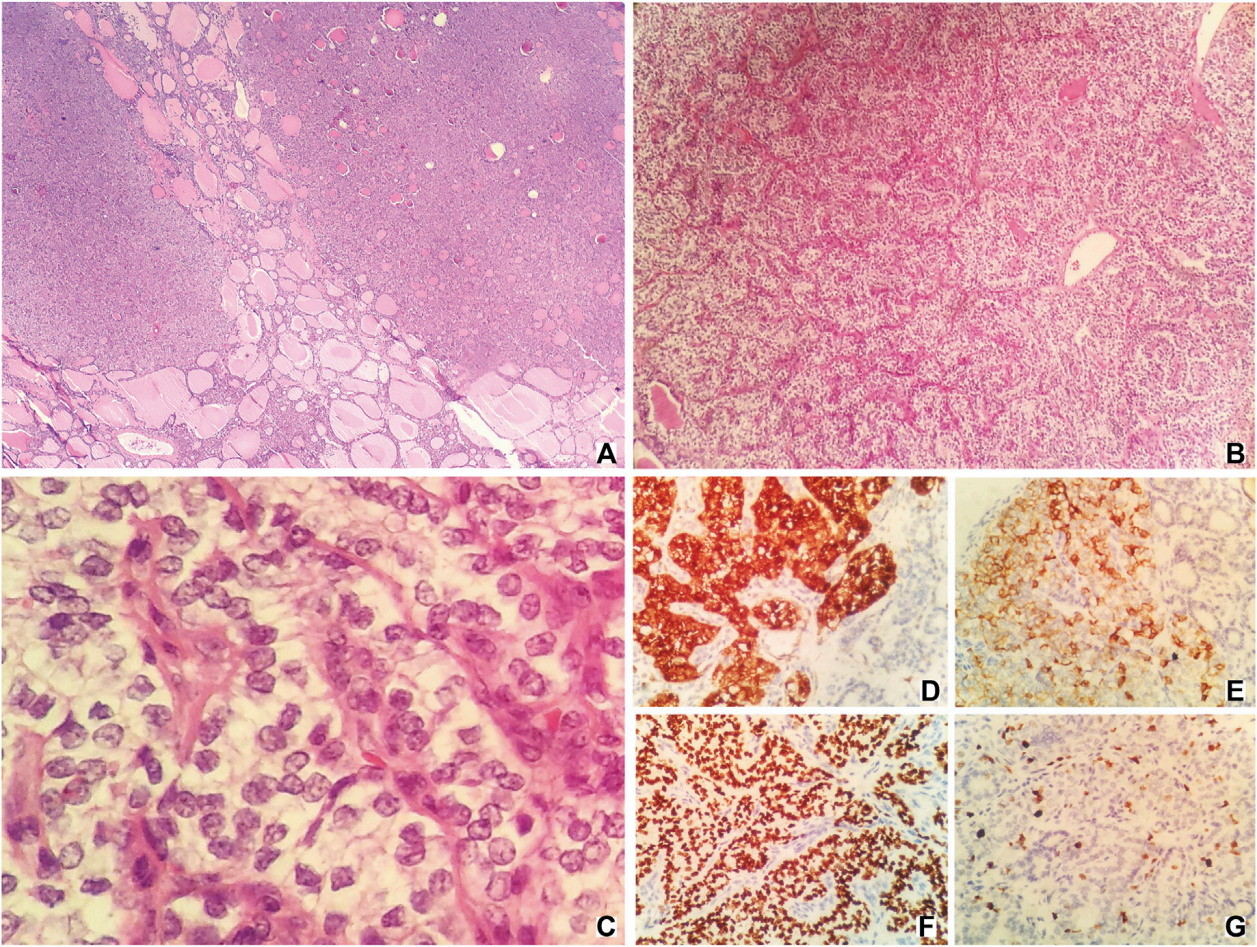
HE staining [**(A–C)**; original magnification ×40, 100, and 400, respectively] and immunohistochemical staining [**(D–G)**; original magnification ×400] of samples taken from the tumor. HE staining **(A,B)** showing the presence of papillae. HE staining c showing irregular, enlarged, ground glass, and overlapping nuclei with grooves, and pseudoinclusions. **(D)**, Galectin-3; **(E)**, CK19; **(F)**, TTF-1; **(G)**, Ki-67.

Pathologic examination after the surgery indicated that the thyroid component could be found in the tissues removed from the right ovary. Hematoxylin-eosin (HE) staining showed that there was a presence of papillae. Irregular, enlarged, ground glass, and overlapping nuclei with grooves and pseudoinclusions could also be found after staining. The tumor was entirely composed of the above component and there was no evidence of benign SO. Immunohistochemically, Galectin-3, CK19 (cytokeratin-19), TTF-1 (thyroid transcription factor-1), and Ki-67 had positive expressions (Figure 2). Tissues of the liver mass, rectal wall nodules, and peritoneal nodules had the same nuclear features as those of the SO (Supplementary Figure S1). Immunohistochemically, these tissues had exactly the same expressions as those of the SO. Also, no abnormality was detected in the thyroid gland and therefore they were considered to be metastatic lesions originated from the SO. Based on findings of HE staining and immunohistochemical staining, as well as clinical recurrence of SO and multiple metastases, the patient was diagnosed with malignant SO (papillary type).

A laboratory test was performed at 1-month follow-up after the surgery and it showed a normal level of FT4 (11.28 pmol/L), TSH (2.518 mIU/L), and Tg (26.50 ng/ml, timeline with relevant serum indicators seen in Supplementary Table S1). In November 2018, 6 months after the laparotomy, a follow-up contrast-enhanced CT examination showed another mass with mixed density and irregular enhancement in the left adnexal region (4.7 × 2.4 cm). Thyroid hormones remained normal. However, the patient refused to accept any other treatment and was since lost to follow up.

## Literature Review

### Materials and Methods

A PubMed literature search of SO case report was performed, with the use of the following terms: “struma ovarii,” “malignant struma ovarii,” and “benign struma ovarii.” A total of 484 articles were retrieved in the search. After selection, 257 eligible articles containing 290 eligible cases were reviewed (including 99 cases with unknown histological behavior, 114 malignant SO cases accounting for 59.69%, and 77 benign SO cases accounting for 40.31%). The search criteria were shown in Supplementary Figure S2. Each variable was stratified by histological behavior. Two-tailed Chi-square test (for discrete variables), two-tailed t test (for continuous variables with a normal distribution), as well as two-tailed K-W test (Kruskal-Wallis test, for continuous variables with a nonnormal distribution) were performed to compare the difference between malignant SO and benign SO. A *p* value <0.05 was considered to be statistically significant. Because there was a third category of SO with unknown histological behavior that could not be classified as benign or malignant, these cases were excluded from further analyses. Also, there were cases with unknown or N/A (not applicable) patient data and these cases were excluded from further analyses to ensure the accuracy of this study.

## Results

**FIGURE 3:**
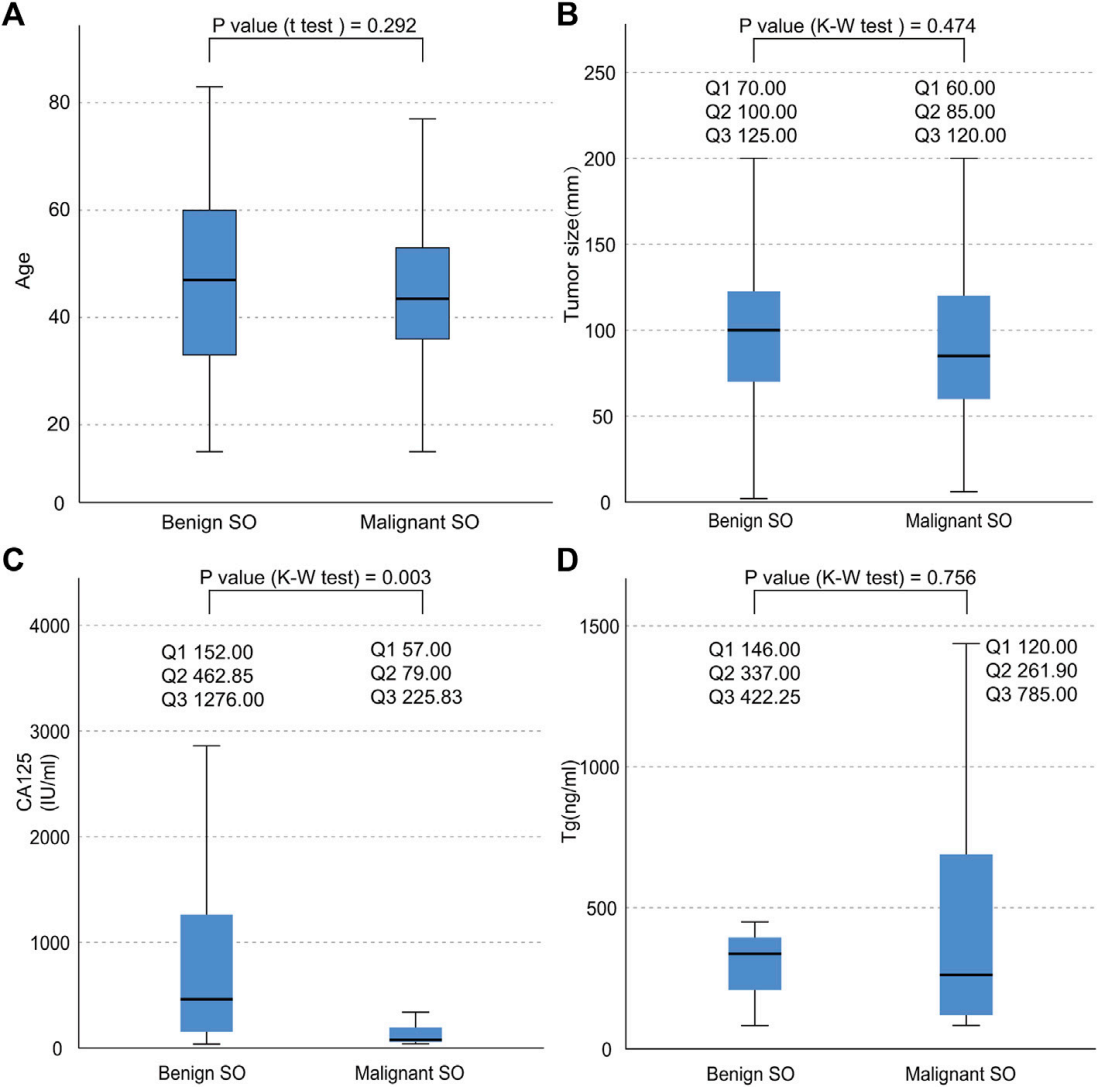
Data distribution of the continuous variables of both benign SO and malignant SO. **(A)**, age distribution; **(B)**, tumor size distribution; **(C)**, serum CA125 distribution; **(D)**, serum Tg distribution; nonnormal variables stated as quartiles; Q1, first quartile; Q2, second quartile; Q3, third quartile.

As shown in Table 1, SO occurred with onset around the age of 47.38 (±16.12) overall. No significant statistical difference was found in terms of age between malignant SO and benign SO (*p* value = 0.292, Figure 3A). The incidence of tumors arising from the right ovary was slightly higher than that arising from the left whether for malignant SO, benign SO, or unknown histological behavior SO, but there was no significant statistical difference between malignant SO and benign SO (*p* value = 0.410). Also, no significant statistical difference was found in tumor size between these two groups (*p* value = 0.474, Figure 3B). For histology, papillary type accounted for the majority of malignant SO (47.62%), whereas follicular type accounted for the majority of benign SO (51.61%) (*p* value = 0.002). For clinical symptoms, no significant statistical differences were found whether in abdominal discomfort, abdominal genital bleeding, or urinary compressive symptom between malignant SO and benign SO (all *p* values > 0.05), but the occurrence of hyperthyroidism was higher for benign SO than that of malignant SO (39.02% vs. 11.94%, *p* value = 0.001). For serum tumor marker CA125, more positive cases were observed in benign SO with a higher serum level than those of malignant SO (all *p* values < 0.05, Table 1; Figure 3C). As for tumor marker Tg, no significant statistical differences were found whether in terms of the positivity rate (percentage of elevated Tg in all malignant or benign SO cases) or serum level between these two groups (all *p* values > 0.05, Table 1; Figure 3D). For the application of surgical methods including salpingo-oophorectomy, pelvic washing, total hysterectomy, and omentectomy, no statistical difference was found between malignant SO and benign SO although benign SO had higher percentages in terms of more aggressive surgical methods. However, more malignant SO cases received total thyroidectomy and RAI (radioactive iodine) treatment than those with benign SO (59.43% vs. 10.71% for total thyroidectomy, 58.04% vs. 11.11% for RAI, all *p* values <0.001). Malignant SO had a higher rate of metastasis than that of benign SO and there was a significant statistical difference (52.94% vs. 15.38%, *p* value = 0.001).

**TABLE 1:** Description of the study population.

	Malignant (%)	Benign (%)	Unknown Behavior (%)	Total (%)	*p* value
Age	45.21 ± 14.30	47.74 ± 17.40	49.59 ± 16.88	47.38 ± 16.12	0.292
Tumor location					0.410
Left ovary	51 (48.11)	32 (42.11)	34 (37.78)	117 (43.01)	
Right ovary	55 (51.89)	43 (56.58)	52 (57.78)	150 (55.15)	
Both ovaries	0 (0)	1 (1.32)	4 (4.44)	5 (1.84)	
Unknown	8	1	9	18	
Histology					0.002
Papillary	50 (47.62)	6 (19.35)	23 (38.98)	79 (40.51)	
Follicular	27 (25.71)	16 (51.61)	17 (28.81)	60 (30.77)	
Follicular variant papillary	21 (20.00)	3 (9.68)	6 (10.17)	30 (15.38)	
Other	7 (6.67)	6 (19.35)	13 (22.03)	26 (13.33)	
Unknown	9	46	40	95	
Abdominal discomfort					0.267
Yes	55 (55.00)	30 (46.15)	37 (45.68)	122 (49.59)	
No	45 (45.00)	35 (53.85)	44 (54.32)	124 (50.41)	
Unknown	14	12	18	44	
Abnormal genital bleeding					0.149
Yes	7 (7.00)	1 (1.54)	8 (9.88)	16 (6.50)	
No	93 (93.00)	64 (98.46)	73 (90.12)	230 (93.50)	
Unknown	14	12	18	44	
Urinary compressive symptom					1.000
Yes	3 (2.97)	2 (3.08)	3 (3.70)	8 (3.24)	
No	98 (97.03)	63 (96.92)	78 (96.30)	239 (96.76)	
Unknown	13	12	18	43	
Hyperthyroidism					0.001
Yes	8 (11.94)	16 (39.02)	15 (31.91)	39 (25.16)	
No	59 (88.06)	25 (60.98)	32 (68.09)	116 (74.84)	
Unknown	47	36	52	135	
Serum CA125					0.001
Positive	16 (42.11)	34 (79.07)	13 (44.83)	63 (57.27)	
Negative	22 (57.89)	9 (20.93)	16 (55.17)	47 (42.73)	
Unknown	76	34	70	180	
Serum Tg					0.867
Positive	29 (51.79)	6 (54.55)	15 (75.00)	50 (57.47)	
Negative	27 (48.21)	5 (45.45)	5 (25.00)	37 (42.53)	
Unknown	58	66	79	203	
Salpingo-oophorectomy					0.093
Unilateral	39 (41.05)	19 (32.20)	24 (31.58)	82 (35.65)	
Bilateral	50 (52.63)	30 (50.85)	38 (50.00)	118 (51.30)	
None	6 (6.32)	10 (16.95)	14 (18.42)	30 (13.04)	
Unknown	7	16	18	41	
N/A	12	2	5	19	
Pelvic washing					0.792
Yes	7 (6.67)	3 (4.48)	3 (3.66)	13 (5.12)	
No	98 (93.33)	64 (95.52)	79 (96.34)	241 (94.88)	
Unknown	9	10	17	36	
Total hysterectomy					0.331
Yes	55 (52.38)	30 (44.78)	31 (38.75)	116 (46.03)	
No	50 (47.62)	37 (55.22)	49 (61.25)	136 (53.97)	
Unknown	7	10	16	33	
N/A	2	—	3	5	
Omentectomy					0.268
Yes	35 (33.33)	17 (25.37)	13 (15.85)	65 (25.59)	
No	70 (66.67)	50 (74.63)	69 (84.15)	189 (74.41)	
Unknown	9	10	17	36	
Total thyroidectomy					<0.001
Yes	63 (59.43)	6 (10.71)	20 (24.69)	89 (36.63)	
No	43 (40.57)	50 (89.29)	61 (75.31)	154 (63.37)	
Unknown	2	10	13	25	
N/A	6	11	5	22	
RAI treatment					<0.001
Yes	65 (58.04)	7 (11.11)	18 (22.22)	90 (35.16)	
No	47 (41.96)	56 (88.89)	63 (77.78)	166 (64.84)	
Unknown	1	10	15	26	
N/A	1	4	3	8	
Metastasis					0.001
Yes	45 (52.94)	4 (15.38)	17 (43.59)	66 (44.00)	
No	40 (47.06)	22 (84.62)	22 (56.41)	84 (56.00)	
Unknown	29	49	60	138	
N/A	—	2	—	2	

N/A, not applicable; CA125, carbohydrate Antigen-125; Tg, thyroglobulin; RAI, radioactive iodine; %, percentage (continuous variables not included); Unknown histological behaviors and unknown or N/A cases of all variables not included in the t test or Chi-square test.

## Discussion

Mature teratomas are composed of tissues from ectodermal, mesodermal, and endodermal origins (7). SO, originated from primordial germ cells of the ovary, is a monodermal and highly specialized mature teratoma and is composed almost entirely of mature thyroid tissue which shows acini filled with thyroid colloid (8). SO includes two forms of histological behavior- malignant and benign. Malignant SO has the same molecular mechanism including BRAF and RAS point mutation and RET/PTC gene rearrangement as that of malignant tumors which originate from the thyroid gland (9, 10). About 5%–15% of ovarian teratomas contain thyroid tissue and they are not necessarily SO. The difference between them is that SO is diagnosed when thyroid tissue is the predominant element. SO, as a variant of ovarian teratoma only accounts for 2% of all ovarian teratomas with thyroid tissue, and approximately only 0.1%–5% of all SO are considered as malignant (11–14). Our study showed that malignant SO accounted for the majority of all SO cases (59.69%) and this result contradicted previous studies. One possible reason is that benign SO is a less rare disease compared with malignant SO, and thus it has been rarely reported. Another, more likely explanation, is that given the lack of clinical complications from benign SO, large series of benign SO are not reported as often as malignant series.

The currently-accepted diagnostic criteria of SO were the one summarized by K Devaney et al. as follows (15, 16): first, the ovarian thyroid tissue must contain cytologically malignant cells and the presence of certain invasion may also contribute to the diagnosis; second, histopathologically, it must exhibit a similar type as that of papillary or follicular carcinoma, sometimes oncocytic or anaplastic carcinoma; third, an immunohistochemically positive Tg expression is also helpful. For papillary type, the diagnosis depends on the appearance of papilla, invasion, and ground glass, empty or overlapping nuclei of the cells, and sometimes along with the existence of psammoma bodies in the tissue.

There has always been controversy over whether cases initially diagnosed as “benign” or “unknown behavior” which developed distant metastases should be classified as malignant SO. Some researchers suggested that malignancy can only be diagnosed when the tumor shows definite invasion or metastases (12, 17), while others diagnose malignancy on nuclear or histologic alterations (including grooves and ground-glass appearance with papillary architecture) (6, 15). Lack of consensus strongly imposes difficulties for diagnosis under this circumstance. Apart from the presence of extraovarian spread, recurrence after initial surgery is another important indication in terms of differential diagnosis, as Shaco-Levy et al. described in their research (18). They subsequently performed another large, blinded study of 86 SO cases aiming to distinguish the pathological disparities between malignant and benign SO. Interestingly, the authors concluded that most pathological features (e.g., papillae, psammoma bodies, nuclear grooves, nuclear overlap, etc) did not correlate with biological behaviors (19). That is, if confined to the ovarian or temporarily “exempted from” relapse, these tumors will not be correctly identified. We agreed that both extraovarian development and recurrence might be more indicative. However, as mentioned above, the establishment of relevant guidelines will surely be needed. On the other hand, we speculated that immunohistochemical findings might also be potentially helpful. For example, some researchers suggested that a positive expression of CK19 is also highly supportive in the diagnosis of malignancy (20), similar to the case in this study. Overall, since this patient showed all the above characteristics, she was diagnosed with malignant SO.

Our study also showed that in general, papillary accounted for the majority of all histological types (40.51%). The study of Wei, S et al., also showed that papillary type was the commonest well-differentiated neoplasm arising in SO (21). This clinical characteristic was similar to that of thyroid carcinoma. However, our study showed that when stratified by histological behavior, papillary type accounted for the majority of malignant SO (47.62%), whereas follicular type accounted for the majority of benign SO (51.61%, Table 1).

Clinical characteristics of SO may include lower abdominal discomfort, abnormal vaginal bleeding and some of those cases may also present ascites, hydrothorax, or hyperthyroidism (22, 23). Our study showed that there were no significant statistical differences in terms of clinical characteristics including abdominal discomfort, abnormal genital bleeding, urinary compressive symptoms between malignant SO and benign SO. Clinically, this might impose difficulties in the differential diagnosis.

According to several reports, only 8% of all cases presented with clinical hyperthyroidism (24, 25). Our study showed a much higher occurrence rate overall (25.16%, Table 1) and benign SO was more likely to develop hyperthyroidism compared with malignant SO (39.02% vs. 11.94%, *p* value = 0.001). We suspected that malignant SO is often poorly differentiated, and thus it rarely develops functioning thyroid tissue. In our case, despite the malignancy, laboratory examination also showed hyperthyroidism. Since no abnormality was detected in ultrasonography when examining the thyroid gland and both FT4 and TSH levels became normal after the surgery, the hyperthyroidism was likely caused by the hyperfunctioning SO (26, 27). Clinically, the diagnosis of SO might be masked by Graves’ disease.

This study showed that the positivity rate of CA125 was about 57.27% in all tumors (including malignant, benign and unknown behavior SO). This result was relatively consistent with previous studies which suggested that the positive value of CA125 was around 60% for all ovarian cancers (28, 29). This suggested that CA125 can not be used as a specific indicator for SO, but it can be helpful in the diagnosis, especially for benign SO with a relatively higher positivity rate (up to 79.07%) and serum level compared with those of malignant SO (Table 1; Figure 3C, all *p* values < 0.05).

Serum Tg may also be a useful indicator in terms of the diagnosis and monitoring of SO. Several studies suggested that the elevated Tg concentration was a useful indicator in the diagnosis and Tg level could be used for detecting the recurrence of SO for patients who received total thyroidectomy (30). Our case study also confirmed the value of this indicator.

Since malignant SO is an extremely rare clinical disease and only a few studies were documented, its therapeutic method has yet to be improved. The therapy, for the time being, ranges largely from radical surgery including total hysterectomy, bilateral salpingo-oophorectomy, and total thyroidectomy followed by postoperative RAI treatment to conservative management including unilateral oophorectomy or preservation of reproductive function and thyroid gland. Chemotherapy may also be useful (5, 31, 32). In our study, no statistical difference was found in terms of the application of salpingo-oophorectomy, pelvic washing, total hysterectomy, and omentectomy between malignant SO and benign SO although malignant SO had higher percentages in terms of more aggressive surgical methods. More malignant SO cases received total thyroidectomy and RAI treatment than those with benign SO. This might be explained by the consideration that malignant SO had a higher metastatic possibility than that of benign SO (52.94% vs 15.38%, *p* value = 0.001). The study of Marti JL, et al. also suggested that thyroidectomy with RAI therapy is reasonable when extra-ovarian spread, metastases, or synchronous primary thyroid gland cancer are present (33).

During the laparoscopic mass dissection in a local primary hospital in August 2008, the mass ruptured. Afterward, another pelvic mass was formed. We suspected that the occurrence of malignant SO might be peritoneal implants at that time, although we could not retrieve more details concerning the pathological confirmation from that surgery. Since malignant SO often has a thin capsule and a large size (first quartile: 60 mm, second quartile: 85 mm, third quartile: 120 mm, Figure 3B) and might be prone to rupture, this might need to be avoided during surgery.

A follow-up contrast-enhanced CT examination showed another recurrent mass (4.7 × 2.4 cm) with mixed density and irregular enhancement in the left adnexal region 6 months after the laparotomy. Although the histology could not be determined because the patient refused any other treatment, we suspected that this might be another recurrent SO. This case report and literature review might be helpful to some extent in the knowledge of this disease.

There were two limitations in this study. First, hyperthyroidism seen in this patient was diagnosed based on the fact that thyroid hormone and TSH levels normalized after resection. However, a ^123^I scan was not performed due to the concerns of avoiding overdiagnosis. Second, analytic results of the literature review only represent cases that have been published to date and they might change to some extent in the future when more cases are added.

## Conclusion

This case report showed that the patient presented with SO repeatedly, which indicated that malignant SO might be prone to relapse. Besides, our literature review also showed that malignant SO often has a high metastatic rate (52.94%). These results suggested that aggressive management including total hysterectomy, bilateral salpingo-oophorectomy, and total thyroidectomy followed by postoperative RAI treatment might need to be recommended for malignant SO. Also, laparotomy might need to be recommended for large tumors that can not be resected by laparoscopic surgery since these tumors might be prone to rupture and thus produce peritoneal implants. Furthermore, a differential diagnosis of other causes of hyperthyroidism might need to be considered.

## Patient Perspective

At 1 month follow-up after the laparotomy, the patient and her family were satisfied with the treatment effect.

## Data Availability

The original contributions presented in the study are included in the article/Supplementary Material, further inquiries can be directed to the corresponding author.

## Supplementary Material

Supplementary Figure S1 HE staining of samples taken from the peritoneal nodules (original magnification × 400).

Supplementary Figure S2 Search criteria of eligible articles and cases.

Supplementary Table S1 Timeline of laboratory tests. CA125, Carbohydrate Antigen-125; Tg, thyroglobulin; FT4, free thyroxine; TSH, thyroid-stimulating hormone.

Supplementary Table S2 Patient’s clinicopathologic parameters. CK19, cytokeratin-19; TTF-1, thyroid transcription factor-1.

## Abbreviations

CA125, carbohydrate antigen-125; CK19, cytokeratin-19; CT, computed tomography; FT4, free thyroxine; K-W test, Kruskal-Wallis test; N/A, not applicable; RAI, radioactive iodine; SO, Struma ovarii; Tg, thyroglobulin; TSH, thyroid-stimulating hormone; TTF-1, thyroid transcription factor-1.
