# Supplementary material for: Case Report: Recurrent Malignant Struma Ovarii With Hyperthyroidism and Metastases, A Rare Case Report and Review of the Literature
Source: Pathol Oncol Res. 2022 May 10;28:1610221. doi: 10.3389/pore.2022.1610221 (PMC9127674; doi:10.3389/pore.2022.1610221)
Supplement: Supplementary file 2 [file Table1.DOCX]

Supplementary Tab.1 Timeline of laboratory tests

|  | April 18th, 2018 | June 25th, 2018 | November 12th, 2018 | Reference range |
| --- | --- | --- | --- | --- |
| CA125 (U/mL) | 118.10 | 12.70 | 10.26 | 0.00-35.00 |
| Tg (ng/mL) | >300 | 26.50 | 20.47 | 0.00-55.00 |
| FT4 (pmol/L) | 22.11 | 11.28 | 10.81 | 9.01-19.05 |
| TSH (uIU/mL) | <0.004 | 2.518 | 2.828 | 0.350-4.940 |

CA125, Carbohydrate Antigen-125; Tg, thyroglobulin; FT4, free thyroxine; TSH, thyroid-stimulating hormone
