# Supplementary figures and images for: Case Report: Recurrent Malignant Struma Ovarii With Hyperthyroidism and Metastases, A Rare Case Report and Review of the Literature
Source: Pathol Oncol Res. 2022 May 10;28:1610221. doi: 10.3389/pore.2022.1610221 (PMC9127674; doi:10.3389/pore.2022.1610221)

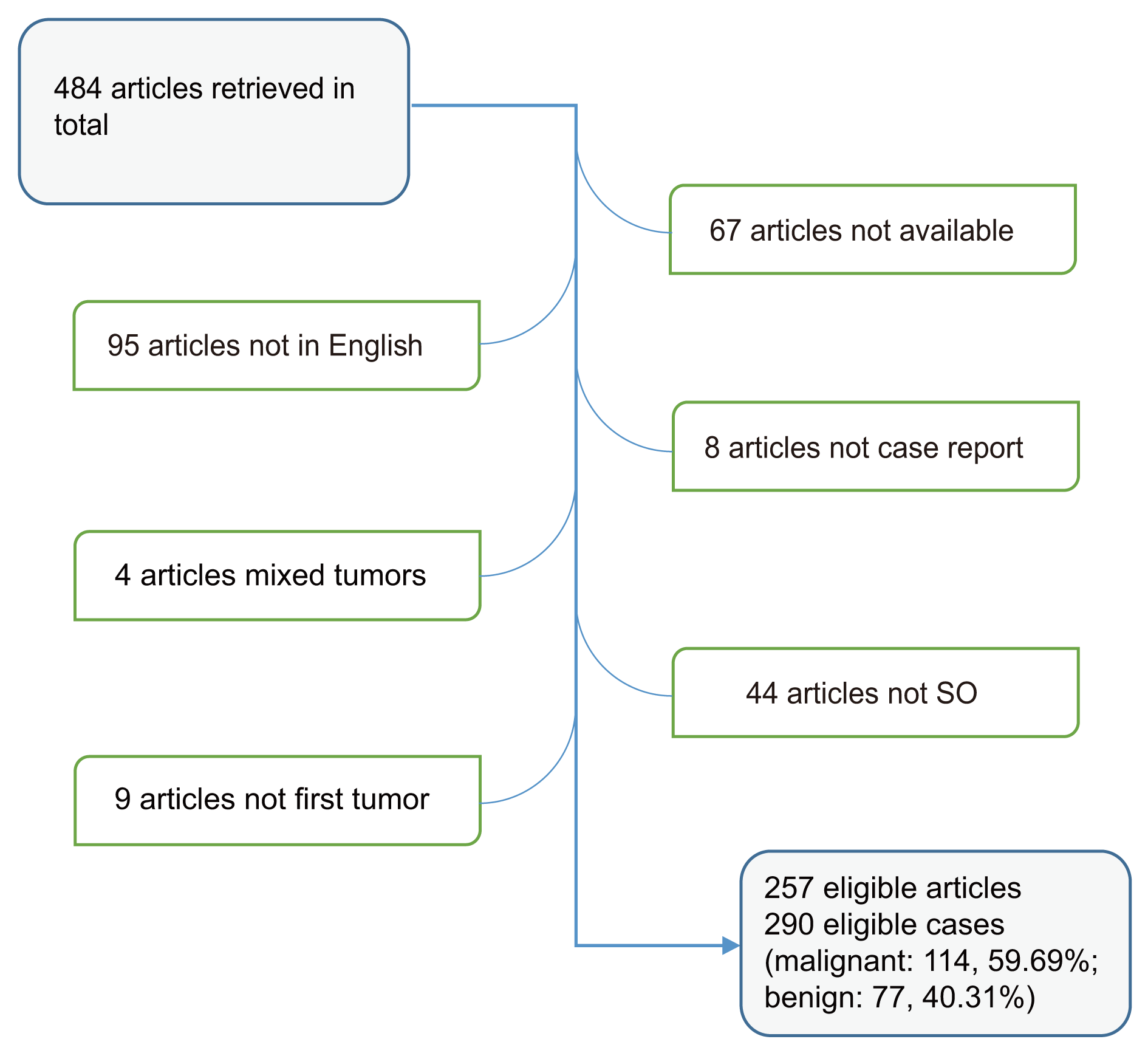

Supplement: Supplementary file 3 [file Image2.TIF]

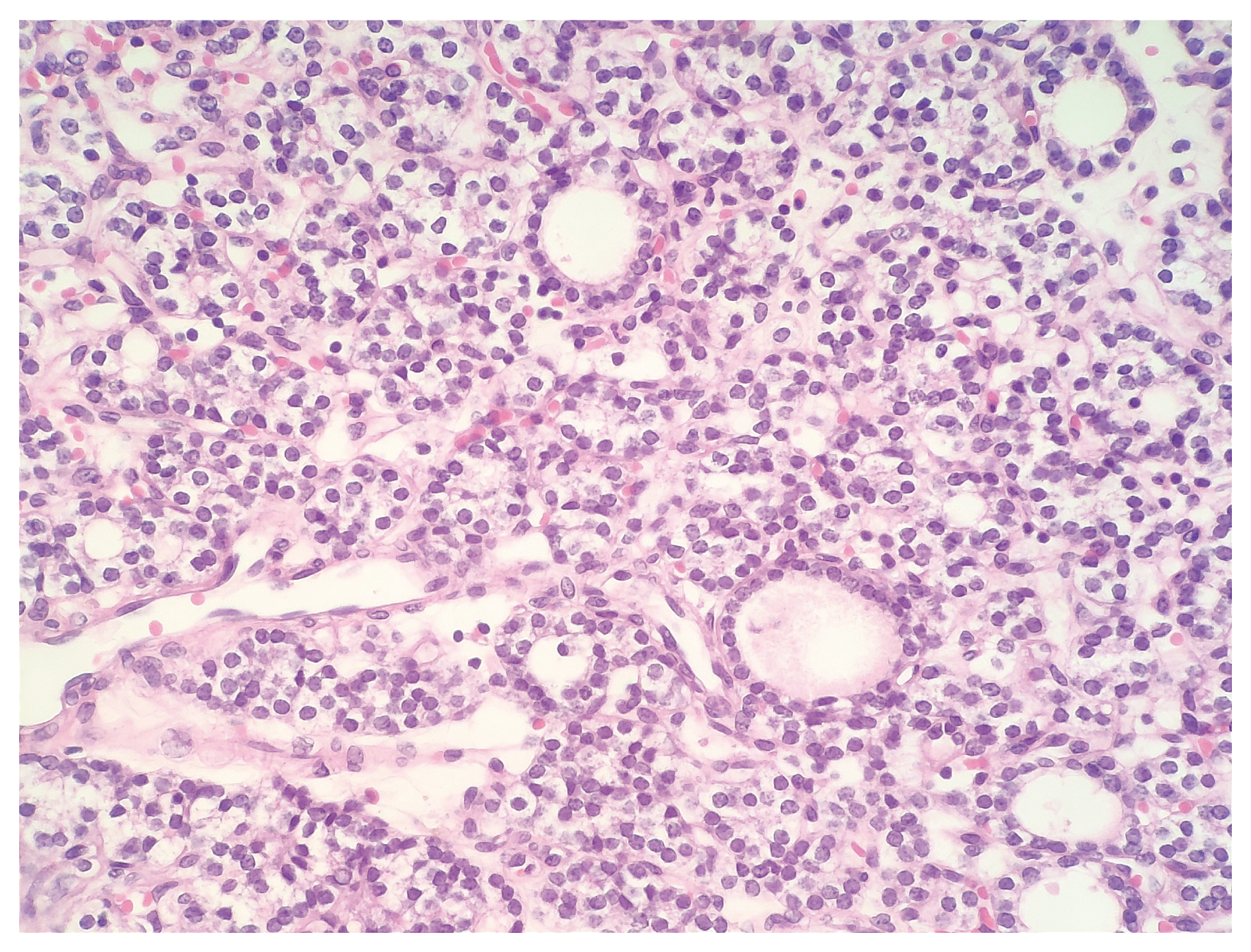

Supplement: Supplementary file 4 [file Image1.TIF]
