# Supplementary material for: Case Report: Recurrent Malignant Struma Ovarii With Hyperthyroidism and Metastases, A Rare Case Report and Review of the Literature
Source: Pathol Oncol Res. 2022 May 10;28:1610221. doi: 10.3389/pore.2022.1610221 (PMC9127674; doi:10.3389/pore.2022.1610221)
Supplement: Supplementary file 5 [file Table2.DOCX]

Supplementary Tab.2 Patient’s clinicopathologic parameters

|  | Results |
| --- | --- |
| Histology type | Papillary |
| Histological behavior | Malignant |
| Galectin-3 | Positive |
| CK19 | Positive |
| TTF-1 | Positive |
| Ki-67 | Positive |

CK19, cytokeratin-19; TTF-1, thyroid transcription factor-1
